# Genome mining for peptidases in heat-tolerant and mesophilic fungi and putative adaptations for thermostability

**DOI:** 10.1186/s12864-018-4549-5

**Published:** 2018-02-20

**Authors:** Tássio Brito de Oliveira, Cene Gostinčar, Nina Gunde-Cimerman, Andre Rodrigues

**Affiliations:** 1 Department of Biochemistry and Microbiology, São Paulo State University (UNESP), Avenida 24-A, 1515, Bela Vista, Rio Claro, SP 13560-900 Brazil; 2 Department of Biology, Biotechnical Faculty, University of Ljubljana, Ljubljana, Slovenia

**Keywords:** Enzyme, Protease, Modeling, Evolution, Thermophilic fungi, Thermotolerant fungi

## Abstract

**Background:**

Peptidases (EC 3.4) consist of a large group of hydrolytic enzymes that catalyze the hydrolysis of proteins accounting for approximately 65% of the total worldwide enzyme production. Peptidases from thermophilic fungi have adaptations to high temperature that makes them adequate for biotechnological application. In the present study, we profiled the genomes of heat-tolerant fungi and phylogenetically related mesophilic species for genes encoding for peptidases and their putative adaptations for thermostability.

**Results:**

We generated an extensive catalogue of these enzymes ranging from 241 to 820 peptidase genes in the genomes of 23 fungi. Thermophilic species presented the smallest number of peptidases encoding genes in relation to mesophilic species, and the peptidases families with a greater number of genes were the most affected. We observed differences in peptidases in thermophilic species in comparison to mesophilic counterparts, at (i) the genome level: a great reduction in the number of peptidases encoding genes that harbored a higher number of copies; (ii) in the primary protein structure: shifts in proportion of single or groups of amino acids; and (iii) in the three-dimensional structure: reduction in the number of internal cavities. Similar results were reported for extremely thermophilic proteins, but here we show for the first time that several changes also occurred on the moderate thermophilic enzymes of fungi. In regards to the amino acids composition, peptidases from thermophilic species in relation to the mesophilic ones, contained a larger proportion of Ala, Glu, Gly, Pro, Arg and Val residues and a lower number of Cys, His, Ile, Lys, Met, Asn, Gln, Ser, Thr and Trp residues (*P* < 0.05). Moreover, we observed an increase in the proportion of hydrophobic and charged amino acids and a decrease in polar amino acids.

**Conclusions:**

Although thermophilic fungi present less genes encoding for peptidases, these have adaptations that could play a role in thermal resistance from genome to protein structure level.

**Electronic supplementary material:**

The online version of this article (10.1186/s12864-018-4549-5) contains supplementary material, which is available to authorized users.

## Background

Isolation and screening of microorganisms have been applied as a strategy to obtain strains able to produce industrially-relevant enzymes. Considering the increased number of available genomes, new rational approaches, such as genome mining, provide an attractive alternative to labor-intense screenings [1, 2]. This is also an interesting alternative to target the prospection of enzymes in fungi deposited in culture collections. Previous successes of genome mining have been documented for lipases [2], lignocellulosic-degrading enzymes [3, 4] and peptidases, particularly in *Aspergillus* species [5].

Peptidases (EC 3.4) consist of a large group of hydrolytic enzymes that catalyze the hydrolysis of proteins by cleavage of the peptide bonds between amino acid residues [6]. The use of microbial peptidases provides technological and economic advantages in industries including detergent, textile, leather, dairy and pharmaceutical production. Peptidases are one of the most important groups of industrial enzymes representing and accounting for approximately 65% of the total enzyme production worldwide [7, 8].

In industrial processes enzymes are often subjected to extreme physicochemical conditions, which are suboptimal for mesophilic ones [1]. Enzymes with potentially unusual properties such as those from thermophilic fungi are thus much sought after. Their enzymes usually have higher thermostability when compared to the mesophilic species, although this is not the case for all proteins. Not only are they of immediate industrial interest but they also enable us to investigate their thermostability patterns and use this knowledge in the rational engineering of thermostability into thermolabile enzymes. Heat-tolerant fungi, often found in composting systems, have been reported as producers of thermostable enzymes with industrial applications [9]. Peptidases from thermophilic fungi have been evaluated in relation to their biochemical properties (e.g. thermal stability) and industrial applications, for instance, *Thermoascus aurantiacus* and its hydrolytic activity on bovine casein [10], *Thermomucor indicae-seudatiacea* and *Rhizomucor miehei* in milk clotting activity [11, 12].

Here, we investigated in silico the diversity of peptidases in the genomes of heat-tolerant fungi and their phylogenetically related mesophilic counterparts. In order to predict the determinants of their thermostability, we investigated the peptidase profile, i.e. catalytic type and families, and amino acid composition of these peptidases and predicted the structural patterns of the representatives from the A1 family aspartic peptidases.

## Methods

### Fungal genomes retrieval and phylogenetic analysis

The annotated genomes of thermophilic, sensu Oliveira et al. [13], thermotolerant and mesophilic species listed in Table 1 were retrieved from public databases, including the National Center for Biotechnology Information (NCBI; http://www.ncbi.nlm.nih.gov/), DOE Joint Genome Institute (JGI, http://genome.jgi.doe.gov/) and Genozyme (http://genome.fungalgenomics.ca/).

**Table 1:** List of fungal genomes mined for peptidase encoding genes

Order	Species	Classification	Reference
Eurotiales	*Aspergillus fumigatus*	Thermotolerant	JGI
	*Aspergillus niger*	Mesophilic	Genozyme
	*Penicillium chrysogenum*	Mesophilic	[49]
	*Penicillium roqueforti*	Mesophilic	[50]
	*Rasamsonia byssochlamydoides*	Thermophilic	Genozyme
	*Talaromyces stipitatus*	Mesophilic	[51]
	*Thermoascus crustaceus*	Thermophilic	Genozyme
	*Thermomyces dupontii*	Thermophilic	Genozyme
	*Thermomyces lanuginosus*	Thermophilic	[52]
	*Thermomyces stellatus*	Thermophilic	Genozyme
Onygenales	*Myceliophthora thermophila*	Thermophilic	[53]
	*Myceliophthora fergusii*	Thermophilic	Genozyme
	*Myceliophthora sepedonium*	Mesophilic	Genozyme
Sordariales	*Thielavia terrestris*	Thermophilic	[53]
	*Thielavia australiensis*	Thermophilic	Genozyme
	*Chaetomium globosum*	Mesophilic	JGI
	*Chaetomium thermophilum*	Thermophilic	[35]
Mucorales	*Mucor circinelloides*	Mesophilic	[54]
	*Rhizomucor pusillus*	Thermophilic	Genozyme
	*Rhizopus delemar*	Mesophilic	[55]
	*Rhizopus microsporus*	Thermotolerant	JGI
	*Thermomucor indicae-seudaticae*	Thermophilic	[3]
Incertae sedis	*Myriococcum thermophilum*	Thermophilic	Genozyme

We inferred a phylogenetic tree to evaluate the evolutionary relationships between the selected species. A super alignment of the selected fungal proteomes was constructed with the Hal pipeline [14], allowing for no missing data. Poorly aligned positions and ones with gaps were removed with Gblocks 0.91b [15]. The following stringent parameters were used: the maximum number of contiguous non-conserved positions was limited to six amino acids, and the minimum length of a block to 15 amino acids. This produced a 106,488-bp-long alignment, which was used for the estimation of the phylogeny. We estimated the best protein evolution model with ProtTest 3.2.1 [16]. The species tree was generated in PhyML 3.3 [17]. We calculated the Approximate Bayes (aBayes) branch supports. The analysis was run using the LG model of evolution. The ProtTest estimate of the α-parameter of the γ-distribution of six substitution rate categories (1.019), and the determined proportion of invariable sites (0.067) were used. The phylogeny data, including alignments, are available in the Treebase repository (http://purl.org/phylo/treebase/phylows/study/TB2:S22179).

### *Thermomucor indicae-seudaticae* genome retrieval and annotation

Few annotated fungal genomes of the order Mucorales were present in the databases. Thus, in the present study we annotated the genome for *T. indicae-seudeticae* (Mucorales: Lichtheimiaceae). The pipeline MAKER was used to annotate the previously unannotated genome of *T. indicae-seudeticae* (GenBank accession number JSYX01.1). Since the transcriptome of this species was not available, we used the following data as evidence to support the annotation in the pipeline: (i) all proteins contained in the MEROPS protease database (downloaded 15. 7. 2016); (ii) all proteins of the Swissprot database (downloaded 15. 7. 2016); and (iii) the transcriptome of *Lichtheimia ramosa* (GenBank GCA_000945115.1), a related species that belongs to the same order. We used three gene predictors in the MAKER pipeline: (i) Semi-HMM-based Nucleic Acid Parser (SNAP) [18], bootstrap-trained within MAKER; (ii) unsupervised-trained GeneMark-ET [19] and (iii) Augustus [20] trained for *Rhizopus oryzae*.

### Search for putative peptidases, thermal adaptation and analysis of the enzymatic profiles in fungi

We mined the proteomes of all investigated fungi for putative protease sequences using the BLAST against the peptidase database MEROPS [21] (http://merops.sanger.ac.uk/). The putative peptidases were classified according to their catalytic site and families by the MEROPS server. An analysis of similarity (ANOSIM) was performed to check for differences in the catalytic type composition between mesophilic and thermophilic species and we applied the Percentage of Similarity analysis (SIMPER) to identify which catalytic type contributes the most to the differences in the enzymatic profile. We conducted the same analyses to evaluate the difference in composition of peptidases families.

The percentage ratio of each type of amino acids and the percentage ratio of charged, polar and hydrophobic amino acids was calculated using the PEPSTATS utility included in the EMBOSS suite. We carried out a paired *t*-test to determine if single amino acid residues or groups contributed to significant differences between thermophilic and mesophilic species in the set of the whole proteins (114,946 and 102,521 proteins, respectively) and the set of peptidases (3340 and 3590 peptidases, respectively); thermotolerant species were not included in the analysis. All analyses were performed in Past v. 2.17c [22]. All results are presented as the changes from mesophilic to thermophilic species.

### Selection of functional homologs and representative proteins from the subfamily A1A aspartic peptidase (AP)

We chose the A1A AP family because it is the most well characterized peptidase. The dataset was scrutinized for the presence of typical AP hallmarks defined as D[TS]G, Y, XXG, D[TS]G, and XXG (where X is any of the hydrophobic residues AFILMV). Sequences lacking any of the hallmarks were considered as non-functional homologues and excluded from further analysis. We made the first alignment manually by the catalytic motif D[TS]G site as described in Revuelta et al. [23]. The second alignment was performed by ClustalW [24].

We performed phylogenetic analysis to identify a cluster of functional sequences in MEGA7 [25]. The evolutionary history was inferred by using the Maximum Likelihood method based on the JTT matrix-based model [26]. Initial tree(s) for the heuristic search were obtained automatically by applying Neighbor-Joining and BioNJ algorithms to a matrix of pairwise distances estimated using a JTT model, and then selecting the topology with superior log likelihood values.

From the initial tree a cluster with 12 protein sequences was selected. This cluster was composed of proteins from *Aspergillus fumigatus*, *A. niger*, *Chaetomium globosum*, *C. thermophilum*, *Myceliophthora fergusii*, *M. sepedonium*, *M. thermophila*, *Myriococcum thermophilum*, *Rasamsonia byssochlamydoides*, *Thermoascus crustaceus*, *Thielavia terrestris* and *T. australiensis*.

### Construction of three-dimensional models by homology

We selected homologous proteins of the family A1A AP as described above. Their amino acid sequences were used to build models using SWISS-MODEL (https://swissmodel.expasy.org/). The SWISS-MODEL template library (SMTL version 2016–09-07, PDB release 2016–09-02) was screened with Blast [27] and HHBlits [28] for evolutionary related structures matching the query sequence. We evaluated the accuracy of each predicted model and its stereo-chemical properties by PROCHECK [29]. The model was selected on the basis of various factors such as overall G-factor, number of residues in core allowed, generously allowed and disallowed regions in Ramachandran plot. We further analyzed the model by QMEAN [30], Lgscore in ProQ [31] and Z-score in ProSA [32].

The sequences were submitted to a bidimensional eletrophoresis in silico using the JVirGel 2.0 [33] to predict the theoretical pI (isoelectric point) and Mw (molecular weight). The number of α-helix, β-strand and β-sheet structures, number of cavities, superficial area and volume were estimated using Swiss-PdbViewer (http://www.expasy.org/spdbv/). We took into consideration that we observed differences in the number of cavities, so we included additional peptidases in the analysis. A total of 50 peptidases were randomly sampled from mesophilic (25) and thermophilic (25) species to the analysis. We tested all the above data with selected proteins from mesophilic and thermophilic species for significance using ANOVA (for continuous data) and Kruskal-Wallis (for counting data).

## Results

### Taxonomic insight into thermophilic fungi

**Fig. 1:**
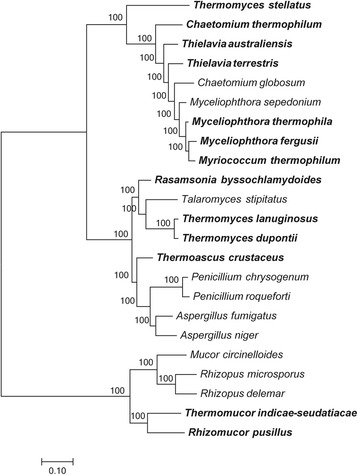
Phylogenetic tree of heat-tolerant fungi and phylogenetically related mesophilic counterparts based on fungal proteomes. Chi2-based branch supports are shown, calculated according to the approximate Likelihood-Ratio Test, as implemented in PhyML 3.3. Thermophilic species are in bold

We compared the genomes of heat-tolerant fungi and their phylogenetically related mesophilic counterparts. A total of 23 species (eight mesophilic, two thermotolerant and 13 thermophilic) were evaluated (Table 1). We inferred a phylogenetic tree using the fungal proteomes to evaluate the evolutionary relationship between the species (Fig. 1). The tree shows that some taxa are not grouped into a monophyletic group, such as *Thermomyces* and *Chaetomium*, indicating that species in these taxa do not belong to the same genus and their phylogenetic position should be reevaluated.

### Peptidases found in fungal genomes

The total number of putative genes encoding for peptidases in the fungal genomes investigated in this study ranged from 241 to 820 (Table 2). Only *Penicillium roqueforti* genome contained all catalytic types (Serine, Aspartic, Metallo, Threonine, Cysteine, Glutamic and Asparagine). *P. roqueforti* has the largest number of putative peptidases (total of 820) followed by *Talaromyces stipitatus* (686), *Rhizopus microsporus* (652), *Myceliophthora sepedonium* (494), *Chaetomium globosum* (469), *Rhizopus delamar* (464), *Aspergillus niger* (437), *Penicillium chrysogenum* (397) and *Mucor circinelloides* (396). These fungi, excepted for *R. microsporus*, are classified as mesophilic (Table 2). Two species of the thermophilic genus *Thermomyces, T. dupontii* and *T. lanuginosus*, contained the smallest number of putative peptidases (241 and 246, respectively).

**Table 2:** Total number of genes encoding for peptidases from heat-tolerant and mesophilic fungal species

Fungi	Putative peptidases	Catalytic type								
		Serine	Aspartic	Metallo	Threonine	Cysteine	Glutamic	Asparagine	Mixed	UNK^a^
Mesophilic										
*Aspergillus niger*	437	204	15	110	24	79	5	0	0	0
*Chaetomium globosum*	469	177	29	123	26	110	4	0	0	0
*Myceliophthora sepedonium*	494	194	29	129	31	106	4	0	1	0
*Mucor circinelloides*	396	108	41	135	21	90	0	0	0	1
*Penicillium chrysogenum*	397	137	33	129	22	73	2	0	0	1
*Penicillium roqueforti*	820	381	36	203	36	146	4	4	0	10
*Rhizopus delemar*	464	122	75	126	34	107	0	0	0	0
*Talaromyces stipitatus*	686	180	164	145	105	82	9	0	0	1
Thermotolerant										
*Aspergillus fumigatus*	349	137	9	101	22	77	2	0	1	0
*Rhizopus microsporus*	652	181	53	212	39	167	0	0	0	0
Thermophilic										
*Chaetomium thermophilum*	277	85	22	74	26	66	4	0	0	0
*Myceliophthora fergusii*	281	85	18	81	23	71	3	0	0	0
*Myceliophthora thermophila*	320	108	23	86	26	73	4	0	0	0
*Myriococcum thermophilum*	318	106	23	82	28	76	3	0	0	0
*Rasamsonia byssochlamydoides*	311	115	16	84	24	70	2	0	0	0
*Rhizomucor pusillus*	347	106	35	109	18	79	0	0	0	0
*Thermoascus crustaceus*	326	122	21	87	23	72	1	0	0	0
*Thermomucor indicae-seudaticae*	297	78	29	101	22	67	0	0	0	0
*Thermomyces dupontii*	241	66	11	71	22	68	4	0	0	0
*Thermomyces lanuginosus*	246	63	14	72	22	70	5	0	0	0
*Thermomyces stellatus*	307	104	14	83	28	76	0	2	0	0
*Thielavia australiensi*^3^	293	89	22	78	26	76	1	0	1	0
*Thielavia terrestris*	340	110	31	84	26	75	5	0	0	0

^a^UNK – Unknown

Asparagine and Glutamic peptidases are not widely distributed among the genomes of fungi explored in the present study. For example *Thermomyces stellatus* and *P. roqueforti* were the only species that presented Asparagine peptidases while Glutamic peptidases were absent in the species belonging to Mucorales. We observed differences between thermophilic and mesophilic species even between closely related ones, e.g. between *Myceliophthora fergusii*, *Myceliophtora thermophila* and *M. sepedonium* (Table 1). The analysis of similarity (ANOSIM) showed that peptidase profiles of mesophilic and thermophilic species differ especially in the number of predicted peptidases (*P* = 0.0001, R = 0.7516). According to the percentage of similarity analysis (SIMPER) the overall peptidase profile between thermophilic and mesophilic species differed by 26.08% (the contribution of each catalytic type is shown in Additional file 1: Table S1).

The entire list of peptidases families and the number of homologous peptidases are shown in Additional file 1: Table S2. From the 138 families of peptidases found, nine are Aspartic peptidases, 32 are Cysteine, one is Glutamic, 52 are Metallo, two are mixed, two are Asparagine, 34 are Serine and six are Threonine. Considering the enzyme families, 11 families contributed to almost 50% of the total difference between thermophilic and mesophilic species (see Additional file 1: Table S3).

### Putative adaptations to thermostability in peptidases

**Fig. 2:**
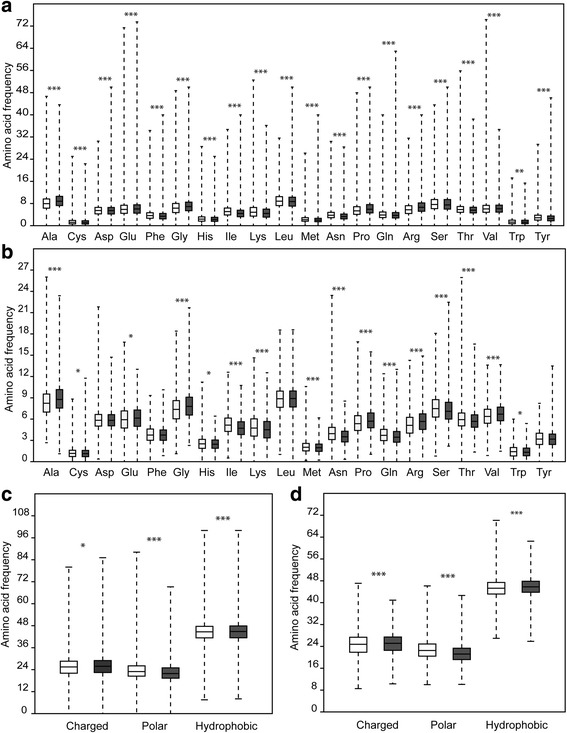
Comparison of amino acid composition between proteins (**a** and **c**) and peptidases (**b** and **d**) from mesophilic (white box plot) and thermophilic (grey box plot) fungi. *P < 0.05; **P < 0.01; ***P < 0.0001

We evaluated the amino acid frequencies in both, the whole proteins in the genome and in the putative proteases. The comparison between the whole proteins in the datasets from mesophilic and thermophilic species showed significant changes in all single and groups of amino acids residues (Fig. 2a and c) in the direction from mesophilic to thermophilic species (*P* < 0.05). We observed an increase in the amino acids Ala, Glu, Gly, Pro, Arg and Val, while a decrease was observed in the other amino acids. The proteins from thermophilic species also showed an increase in charged and hydrophobic residues and a decrease in polar residues.

Regarding the peptidases, we noted the same pattern observed for all proteins of thermophilic species (*P* < 0.05), such as an increasing in the amino acids Ala, Glu, Gly, Pro, Arg and Val, an increase in charged and hydrophobic residues, and a decrease in polar residues (Fig. 2b and d). On the other hand, we observed a decrease in the amino acids Cys, His, Ile, Lys, Met, Asn, Gln, Ser, Thr, Trp and no differences were found for Asp, Phe, Leu, and Tyr residues. A detailed table with statistical data is available in Additional file 1: Table S4.

To evaluate characteristics in the three-dimensional structures of orthologous peptidases, we built 3D models of A1A AP peptidases, one of the most characterized family. They were evaluated via Procheck, QMEAN, ProSa (Z-score) and ProQ (Lgscore) and the values support the models shown in Additional file 1: Table S4. The stereo-chemical quality of the model structures showed that the majority of amino acids are in the most favored and additionally allowed favored regions (Additional file 1: Table S5). No significant differences were found in the number of α-helix, β-strand and β-sheet, superficial area, volume, molecular weight and isoeletric point. On the other hand, the number of cavities decreased in peptidases from thermophiles (*P* = 0.0185, Kruskal-Wallis test) (Table 3). Although the proteins presented differences in amino acid composition, the conformational structure is the same, maintaining the basic structure of the family A1A (Additional file 1: Figure S1).

**Table 3:** Characterization of the Aspartic Peptidase protein and the three-dimensional structure

Fungi	Mol. Mass (Kda)	pI	α-helix	β-strands	β -sheet	Superficial area (Å^2^)	Volume (Å^3^)	N° of cavities
Non-thermophilic								
*Aspergillus fumigatus*	49.99	5.88	6	25	3	12.76	44.29	8
*Aspergillus niger*	46.72	4.56	6	27	4	12.67	43.50	5
*Chaetomium globosum*	80.29	5.52	7	25	3	13.44	43.52	5
*Myceliophthora sepedonium*	52.07	4.67	6	24	3	13.09	44.33	6
Thermophilic								
*Chaetomium thermophilum*	49.38	7.82	3	25	3	13.49	43.72	5
*Myceliophthora fergusii*	52.44	4.57	7	24	3	13.57	44.01	3
*Myceliophthora thermophila*	52.45	4.39	7	24	3	13.57	44.01	3
*Myriococcum thermophilum*	52.08	4.84	5	25	3	13.45	44.10	4
*Rasamsonia bysoclamydoides*	45.41	4.73	8	26	3	12.45	42.15	4
*Thermoascus crustaceus*	47.43	4.89	8	26	3	13.26	43.40	5
*Thielavia australiensis*	45.57	7.22	6	25	4	12.89	43.62	2
*Thielavia terrestris*	74.85	5.13	7	25	3	13.21	42.21	3

**Fig. 3:**
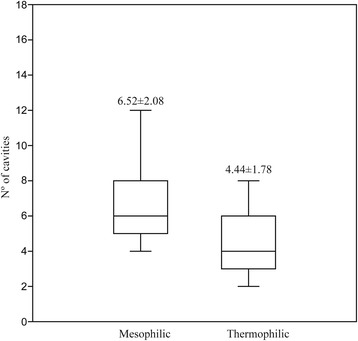
Differences between the number of cavities in peptidases from mesophilic and thermophilic fungi. Different letters represent statistical difference (*P* = 0.0009441, Kruskal-Wallis test)

Due to the observed difference in the number of cavities, we evaluated additional peptidases from the family A1A (a total of 50 peptidases) to confirm this pattern. A significant difference (*P* = 0.0009441, Kruskal-Wallis test) was observed in the peptidases from mesophilic and thermophilic species (6.52 ± 2.08 and 4.44 ± 1.78, respectively), confirming our previous findings (Fig. 3).

## Discussion

Genome streamlining by genome reduction has been reported to prokaryotic organisms as negatively correlated to growth temperature [34]. Thermophilic fungi have also experienced a genome reduction in response to thermal adaptation and consequently they lost many genes during their evolution [35], among them, the peptidases coding-genes, as shown for the first time in this study. Our results showed the largest reduction in the peptidases families with a higher number of genes, while those with fewer or single copies were less affected. The observed reduction is in contrast with the observations for cellulolytic enzymes, which were expanded in thermophilic fungal genomes but there was no mention regarding the peptidases-coding genes and how they are affected with this reduction [35].

Thermostable peptidases acting at high temperatures (65–85 °C) have already been applied in the baking, brewing, detergent and leather industries [36]. Thermophilic fungi are recognized as an interesting source of hydrolytic enzymes with industrial application, for example amylases, cellulases, hemicellulases, lipases and peptidases [9]. Despite the reduction in the number of copies of peptidases-coding genes, here we report a large catalogue of these enzymes, providing a good basis for further investigation and application.

A promising strategy to improve thermostability in proteins is the site-directed mutagenesis [37]. However, there is no consensus about the relationship of amino acid composition and its role in thermal adaptation. Increases in charged or hydrophobic residues, or both, are often reported, but their contribution to thermostability is still a topic of discussion [38].

Although we confirmed some of the previously observed changes in amino acid composition in our peptidases dataset (e.g. increased hydrophobic and charged residues), some of our observations differ from previous reports on thermal adaptations. These differences include observed increase in Trp [35, 39], lower frequency of Asp in eukaryotic proteins [35], an increase in Tyr and Ile and less Glu and Arg in M4 peptidases in prokaryotes species [40] all of which were not confirmed in our study. These observations suggest that while amino acid substitutions in thermoadaptation follow some general patterns, there are specific adaptations that differ between Archaea, Bacteria and Eukarya. These differences are observed even for different groups of proteins, as detected in the peptidases evaluated in this study when compared to the other proteins in the same genomes. It warrants the need to study thermoadaptation on a case-to-case basis.

The increase in Ala, Glu, Gly, Pro, Arg and Val content of peptidases from thermophilic fungi are in line with reports that some of these amino acids increase thermostability of proteins. They can improve the thermal stability by (i) forming a large number of electrostatic interactions (e.g. hydrogen bond and salt bridges), such as is the case of Glu and Arg [41, 42], (ii) increasing the rigidity of proteins, such as by the cyclic structure in the side chain of Pro [42], (iii) maintaining hydrophobic pockets, e.g. with Ala [43], or (iv) increasing the number of weak interactions, e.g. with Gly [44].

On the other hand, other amino acids that had a content decrease are known to reduce thermal stability, as Met and Asn, by the chemical instability of these residues at high temperatures [39]. Asn and Gln deaminate easily and Cys is susceptible to oxidation at elevated temperature [45]. Unless it is required for activity or formation of disulfide bonds, Cys is often absent from thermophilic proteins [45].

Gly and Pro residues have a major influence on the kinetics of loop formation in proteins. Glycine accelerates loop formation by decreasing the activation energy and it is known to contribute to conformational flexibility of polypeptide chains and to flexibility of some loops associated with enzymatic catalysis [46, 47]. Cis Prolyl shows the fastest kinetics of all sequences despite an increased activation energy [46]. The frequency of Pro in the modeled proteins was increased in the proteases from thermophilic fungi mainly in the loop areas. Although the increase of Pro is often seen in organisms with high GC content, in thermophilic fungi this content does not differ significantly between mesophilic and thermophilic species [35].

Although we observed significant differences between the amino acid composition of peptidases from thermophilic and mesophilic fungi, their predicted structures remained relatively unchanged. The only exception was the significant decrease in the number of cavities in peptidases from thermophiles. In addition to the observed reduction in the number of peptidases, we also observed the possible effect of natural selection. In this sense, there are two possible evolutionary scenarios: i) thermophilic fungi have lost peptidases with large number of cavities and kept only those that are compactly folded or ii) peptidases from thermophilic fungi were optimized to contain fewer cavities.

In general, cavities are considered as packing defects destabilizing the native structure [48]. The peptidases of thermophilic fungi present the same adaptation observed in thermophilic enzymes and it was interpreted as an adaptation for protein thermostability [39]. However, the low number of cavities was only observed for hyperthermophilic enzymes but in this study we report for moderate thermophilic enzymes as well.

## Conclusions

Although thermophilic fungi present less genes encoding for peptidases, they have adaptations that could play a role in thermal resistance. These can occur from the genome to the protein structure level. Exploring the patterns that improve thermal stability in specific proteins can accelerate the process of finding species able to produce enzymes with the desired properties. This strategy combined with genome mining can drive the selection of target enzymes with characteristics indicating higher thermal stability. Moreover, this approach can find patterns to improve mesophilic proteins by site-directed mutagenesis for engineering enzymes adapted to high temperatures. Our results are not only of biotechnological interest but they also have an evolutionary appeal. In addition, the results prompt hypotheses on the structural differences related to temperature and stability that can be experimentally tested.

## Additional file

Additional file 1: **Table S1** Contribution of the seven catalytic types for the differences between peptidases of thermophilic and mesophilic species as shown by Percentage of similarity analysis (SIMPER). **Table S2** Catalogue of peptidases in thermophilic, thermotolerant and mesophilic fungal genomes. **Table S3** Peptidase families that most contribute (Cumulative > 50% of contribution) to the differences between thermophilic and mesophilic fungi as shown by analysis of percentage of similarity (SIMPER). **Table S4** Differences between the number of cavities in peptidases from mesophilic and thermophilic fungi. The differences were tested using the T-test, with *n*-1 degrees of freedom, for a total of 102,521 and 114,946 proteins and 3590 and 3340 peptidases from mesophilic and thermophilic fungi, respectively. **Table S5** Validation parameters computed for built 3D protein of the Aspartic peptidase sequence. **Figure S1** Predicted three-dimmensional structures of selected aspartic acid peptidases of fungi. (A) *Aspergillus fumigatus*; (B) *A. niger*; (C) *Chaetomim globosum*; (D) *C. thermophilum*; (E) *Myceliophthora fergusii*; (F) *M. sepedonium*; (G) *M. thermophila*; (H) *Myriococcum thermophilum*; (I) *Rasamsonia bycochlamydoides*; (J) *Thermoascus crustaceus*; (K) *Thielavia australiensis*; and (L) *T. terrestris*. (DOCX 3863 kb)
