# Supplementary material for: Genome mining for peptidases in heat-tolerant and mesophilic fungi and putative adaptations for thermostability
Source: BMC Genomics. 2018 Feb 20;19:152. doi: 10.1186/s12864-018-4549-5 (PMC5819190; doi:10.1186/s12864-018-4549-5)

**SUPPLEMENTARY MATERIAL**

# Genome mining for peptidases in heat-tolerant and mesophilic fungi and putative adaptations for thermostability

Tássio Brito de Oliveira, Cene Gostinčar, Nina Gunde-Cimerman and Andre Rodrigues

**Addition file 1**. Contribution of the seven catalytic types for the differences between peptidases of thermophilic and mesophilic species as shown by Percentage of similarity analysis (SIMPER)

| **Catalytic type** | **Contrib. %** | **Cumulative %** |
| --- | --- | --- |
| Serine | 40.58 | 40.58 |
| Metallo | 24.34 | 64.91 |
| Aspartic | 14.72 | 79.63 |
| Cysteine | 12.17 | 91.8 |
| Threonine | 6.22 | 98.02 |
| Glutamic | 1.23 | 99.25 |
| Unknown | 0.43 | 99.68 |
| Asparagine | 0.23 | 99.91 |
| Mixed | 0.07 | 100 |

**Additional file 2.** Catalogue of peptidases in thermophilic, thermotolerant and mesophilic fungal genomes.

| **Peptidase**  **Family/**  **Subfamily** |  |  |  |  |  | **Fungal species** | | | | | | | | | | | | | | | | | | |
| --- | --- | --- | --- | --- | --- | --- | --- | --- | --- | --- | --- | --- | --- | --- | --- | --- | --- | --- | --- | --- | --- | --- | --- | --- |
|  | **Enzyme type** | *Aspergillus niger* | *Aspergillus fumigatus* | *Myceliophthora sepedonium* | *Rhizopus delamar* | *Mucor circinelloides* | *Chaetomium globosum* | *Penicillium chrysogenum* | *Penicillium roqueforti* | *Talaromyces stipitatus* | *Rhizopus microsporus* | *Chaetomium thermophilum* | *Myceliophthora fergusii* | *Myceliophthora thermophila* | *Myriococcum thermophilum* | *Rasamsonia byssochlamydoides* | *Rhizomucor pusillus* | *Thermoascus crustaceus* | *Thermomyces dupontii* | *Thermomyces lanuginosus* | *Thermomyces stellatus* | *Thielavia australiensis* | *Thielavia terrestris* | *Thermomucor indicae-seudaticae* |
| A1A | pepsin A | 12 | 7 | 26 | 44 | 37 | 22 | 11 | 11 | 21 | 40 | 19 | 15 | 20 | 20 | 11 | 31 | 16 | 7 | 7 | 11 | 18 | 27 | 25 |
| A1B | Nepenthesin | 0 | 0 | 0 | 0 | 0 | 0 | 0 | 0 | 0 | 0 | 0 | 0 | 0 | 0 | 1 | 0 | 0 | 0 | 0 | 0 | 0 | 0 | 0 |
| A1 | not assigned to subfamily | 0 | 0 | 0 | 1 | 0 | 0 | 0 | 3 | 0 | 0 | 0 | 0 | 1 | 0 | 0 | 0 | 0 | 0 | 0 | 0 | 0 | 0 | 0 |
| A2A | HIV-1 retropepsin | 0 | 0 | 0 | 2 | 0 | 0 | 0 | 6 | 0 | 0 | 0 | 0 | 0 | 0 | 0 | 0 | 0 | 0 | 0 | 0 | 1 | 0 | 0 |
| A11A | Copia transposon peptidase | 0 | 0 | 0 | 0 | 0 | 2 | 19 | 10 | 140 | 0 | 0 | 0 | 0 | 0 | 1 | 0 | 0 | 1 | 4 | 0 | 0 | 0 | 0 |
| A22B | impas 1 peptidase | 2 | 1 | 2 | 1 | 2 | 4 | 2 | 1 | 2 | 3 | 2 | 2 | 2 | 2 | 2 | 1 | 2 | 2 | 2 | 2 | 2 | 3 | 1 |
| A22A | presenilin 1 | 0 | 0 | 0 | 1 | 2 | 0 | 0 | 2 | 0 | 3 | 0 | 0 | 0 | 0 | 0 | 2 | 0 | 0 | 0 | 0 | 0 | 0 | 2 |
| A28A | DNA-damage inducible protein 1 | 1 | 1 | 1 | 24 | 0 | 1 | 0 | 1 | 0 | 7 | 1 | 1 | 0 | 1 | 1 | 1 | 3 | 1 | 1 | 1 | 1 | 1 | 1 |
| A33 |  | 0 | 0 | 0 | 0 | 0 | 0 | 1 | 0 | 1 | 0 | 0 | 0 | 0 | 0 | 0 | 0 | 0 | 0 | 0 | 0 | 0 | 0 | 0 |
| C1A | Papain | 0 | 1 | 0 | 1 | 0 | 1 | 1 | 5 | 0 | 1 | 0 | 0 | 3 | 0 | 0 | 0 | 0 | 0 | 1 | 1 | 0 | 1 | 0 |
| C2A | calpain-2 | 3 | 3 | 8 | 1 | 2 | 6 | 3 | 2 | 2 | 2 | 5 | 4 | 4 | 6 | 3 | 1 | 3 | 2 | 3 | 5 | 6 | 6 | 2 |
| C1B | bleomycin hydrolase | 1 | 0 | 2 | 1 | 1 | 0 | 0 | 0 | 1 | 2 | 0 | 1 | 1 | 2 | 1 | 1 | 1 | 1 | 1 | 1 | 1 | 0 | 1 |
| C11 | kyphoscoliosis peptidase | 2 | 2 | 2 | 0 | 0 | 2 | 0 | 3 | 0 | 0 | 2 | 2 | 2 | 2 | 2 | 0 | 2 | 2 | 2 | 2 | 2 | 2 | 0 |
| C12 | ubiquitinyl hydrolase-L1 | 4 | 4 | 7 | 5 | 7 | 8 | 4 | 4 | 4 | 10 | 3 | 2 | 4 | 5 | 3 | 5 | 3 | 3 | 3 | 4 | 5 | 5 | 3 |
| C13 | Legumain | 1 | 1 | 1 | 1 | 1 | 1 | 1 | 3 | 1 | 3 | 1 | 1 | 1 | 1 | 1 | 1 | 1 | 1 | 1 | 1 | 1 | 1 | 2 |
| C14B | metacaspase Yca1 | 5 | 2 | 4 | 10 | 5 | 8 | 3 | 6 | 5 | 6 | 2 | 3 | 2 | 4 | 2 | 2 | 3 | 2 | 2 | 3 | 2 | 2 | 3 |
| C15 | pyroglutamyl-peptidase I | 0 | 1 | 2 | 0 | 0 | 2 | 1 | 1 | 1 | 0 | 1 | 2 | 1 | 1 | 1 | 0 | 0 | 0 | 1 | 1 | 1 | 1 | 0 |
| C19 | ubiquitin-specific peptidase 14 | 26 | 27 | 36 | 40 | 37 | 33 | 21 | 37 | 29 | 75 | 22 | 22 | 23 | 23 | 27 | 27 | 25 | 29 | 28 | 28 | 24 | 23 | 22 |
| C25 | gingipain RgpA | 0 | 0 | 0 | 0 | 0 | 0 | 0 | 1 | 0 | 0 | 0 | 0 | 0 | 0 | 0 | 0 | 0 | 0 | 0 | 0 | 0 | 0 | 0 |
| C26 | gamma-glutamyl hydrolase | 13 | 12 | 16 | 15 | 6 | 14 | 14 | 34 | 13 | 19 | 11 | 13 | 12 | 12 | 11 | 14 | 12 | 11 | 10 | 11 | 13 | 11 | 13 |
| C33 | equine arteritis virus Nsp2-type cysteine peptidase | 0 | 0 | 0 | 1 | 0 | 0 | 0 | 0 | 0 | 0 | 0 | 0 | 0 | 0 | 0 | 0 | 0 | 0 | 0 | 0 | 0 | 0 | 0 |
| C40 | dipeptidyl-peptidase VI | 0 | 1 | 0 | 0 | 0 | 0 | 1 | 4 | 0 | 0 | 0 | 0 | 0 | 0 | 0 | 0 | 0 | 0 | 0 | 0 | 0 | 0 | 0 |
| C44 | amidophosphoribosyltransferase precursor | 8 | 4 | 8 | 8 | 11 | 9 | 7 | 7 | 5 | 7 | 5 | 6 | 5 | 7 | 5 | 6 | 6 | 4 | 5 | 5 | 6 | 5 | 6 |
| C45 | acyl-coenzyme A:6-aminopenicillanic acid acyl-transferase precursor | 1 | 1 | 0 | 0 | 0 | 0 | 3 | 1 | 3 | 0 | 0 | 0 | 0 | 0 | 1 | 0 | 2 | 0 | 0 | 1 | 0 | 1 | 0 |
| C48 | Ulp1 peptidase | 3 | 4 | 4 | 8 | 5 | 7 | 4 | 4 | 6 | 6 | 4 | 4 | 4 | 4 | 3 | 5 | 4 | 3 | 3 | 3 | 3 | 6 | 3 |
| C50 | Separasse | 0 | 2 | 4 | 1 | 1 | 2 | 1 | 4 | 2 | 2 | 1 | 2 | 1 | 0 | 2 | 1 | 2 | 1 | 1 | 1 | 3 | 2 | 0 |
| C51 | D-alanyl-glycyl peptidase | 0 | 0 | 0 | 0 | 0 | 0 | 0 | 0 | 0 | 0 | 0 | 1 | 0 | 0 | 0 | 0 | 0 | 0 | 0 | 0 | 0 | 0 | 0 |
| C54 | autophagin-1 | 1 | 1 | 1 | 2 | 2 | 2 | 1 | 3 | 1 | 4 | 1 | 1 | 1 | 1 | 1 | 1 | 1 | 1 | 1 | 1 | 1 | 1 | 2 |
| C56 | PfpI peptidase | 5 | 6 | 4 | 3 | 3 | 9 | 3 | 16 | 5 | 7 | 3 | 2 | 4 | 2 | 2 | 3 | 2 | 2 | 2 | 3 | 3 | 3 | 2 |
| C60 | Sortase | 0 | 0 | 0 | 0 | 0 | 0 | 0 | 1 | 0 | 0 | 0 | 0 | 0 | 0 | 0 | 0 | 0 | 0 | 0 | 0 | 0 | 0 | 0 |
| C65 | otubain-1 | 1 | 1 | 1 | 0 | 1 | 2 | 1 | 1 | 1 | 4 | 1 | 1 | 1 | 1 | 1 | 1 | 1 | 1 | 1 | 1 | 1 | 1 | 0 |
| C67 | CylD peptidase | 0 | 0 | 0 | 1 | 0 | 0 | 0 | 0 | 0 | 0 | 0 | 0 | 0 | 0 | 0 | 1 | 0 | 0 | 0 | 0 | 0 | 0 | 0 |
| C69 | dipeptidase A | 0 | 0 | 0 | 0 | 0 | 0 | 0 | 0 | 0 | 0 | 0 | 0 | 0 | 0 | 0 | 0 | 0 | 0 | 0 | 1 | 0 | 0 | 0 |
| C78 | UfSP1 peptidase | 0 | 0 | 0 | 0 | 0 | 0 | 0 | 1 | 0 | 1 | 0 | 0 | 0 | 0 | 0 | 2 | 0 | 0 | 0 | 0 | 0 | 0 | 0 |
| C82A | L,D-transpeptidase | 0 | 0 | 0 | 0 | 0 | 0 | 0 | 0 | 0 | 0 | 0 | 0 | 0 | 0 | 0 | 0 | 0 | 1 | 1 | 0 | 0 | 0 | 0 |
| C83 | gamma-glutamylcysteine dipeptidyltranspeptidase | 0 | 0 | 0 | 3 | 2 | 0 | 0 | 0 | 0 | 4 | 0 | 0 | 0 | 0 | 0 | 2 | 0 | 0 | 0 | 0 | 0 | 0 | 2 |
| C85A | OTLD1 deubiquitinylating enzyme | 2 | 2 | 2 | 1 | 2 | 0 | 2 | 3 | 1 | 7 | 1 | 1 | 1 | 1 | 2 | 2 | 2 | 2 | 2 | 1 | 1 | 1 | 2 |
| C85B | OTU1 peptidase | 1 | 1 | 1 | 1 | 0 | 2 | 0 | 0 | 0 | 2 | 1 | 1 | 1 | 1 | 1 | 1 | 1 | 0 | 1 | 0 | 1 | 1 | 1 |
| C86 | ataxin-3 | 0 | 0 | 0 | 0 | 1 | 0 | 0 | 0 | 0 | 0 | 0 | 0 | 0 | 0 | 0 | 1 | 0 | 0 | 0 | 0 | 0 | 0 | 1 |
| C88 |  | 0 | 0 | 0 | 0 | 1 | 0 | 1 | 0 | 01 | 0 | 0 | 0 | 0 | 0 | 0 | 0 | 0 | 0 | 0 | 0 | 0 | 0 | 0 |
| C97 | DeSI-1 peptidase | 1 | 1 | 3 | 4 | 0 | 4 | 0 | 3 | 0 | 5 | 1 | 2 | 2 | 2 | 1 | 2 | 1 | 1 | 1 | 2 | 2 | 2 | 2 |
| G1 | scytalidoglutamic peptidase | 5 | 2 | 4 | 0 | 0 | 4 | 2 | 3 | 9 | 0 | 4 | 3 | 4 | 3 | 2 | 0 | 1 | 4 | 5 | 0 | 1 | 5 | 0 |
| M1 | aminopeptidase N | 4 | 4 | 5 | 6 | 5 | 9 | 5 | 4 | 4 | 11 | 3 | 3 | 4 | 4 | 4 | 5 | 4 | 4 | 4 | 3 | 4 | 4 | 3 |
| M3A | thimet oligopeptidase | 6 | 3 | 12 | 3 | 3 | 6 | 6 | 5 | 6 | 6 | 3 | 5 | 5 | 4 | 5 | 3 | 3 | 3 | 3 | 5 | 3 | 5 | 3 |
| M4 | Thermolysin | 0 | 0 | 1 | 0 | 0 | 0 | 0 | 0 | 0 | 0 | 1 | 0 | 0 | 0 | 0 | 0 | 1 | 0 | 0 | 1 | 0 | 0 | 0 |
| M6 | immune inhibitor A peptidase | 0 | 0 | 0 | 0 | 0 | 0 | 1 | 0 | 0 | 0 | 0 | 0 | 0 | 0 | 0 | 0 | 0 | 0 | 0 | 0 | 0 | 1 | 0 |
| M10B | Serralysin | 1 | 0 | 0 | 0 | 0 | 0 | 0 | 0 | 0 | 0 | 0 | 0 | 0 | 0 | 0 | 0 | 0 | 0 | 0 | 0 | 0 | 1 | 0 |
| M12B | Adamalysin | 2 | 3 | 1 | 3 | 3 | 0 | 2 | 2 | 1 | 2 | 1 | 1 | 1 | 1 | 1 | 3 | 1 | 0 | 1 | 0 | 1 | 1 | 2 |
| M12 | not assigned to subfamily | 0 | 1 | 0 | 1 | 0 | 0 | 0 | 0 | 0 | 3 | 0 | 0 | 0 | 0 | 0 | 0 | 0 | 1 | 0 | 2 | 0 | 0 | 0 |
| M13 | Neprilysin | 0 | 1 | 1 | 9 | 8 | 0 | 1 | 36 | 1 | 13 | 0 | 0 | 0 | 0 | 1 | 5 | 1 | 0 | 0 | 1 | 0 | 2 | 5 |
| M14A | carboxypeptidase A1 | 2 | 1 | 5 | 0 | 0 | 5 | 2 | 2 | 1 | 0 | 4 | 3 | 3 | 3 | 1 | 0 | 1 | 1 | 1 | 2 | 3 | 2 | 0 |
| M16A | Pitrilysin | 8 | 4 | 6 | 7 | 6 | 8 | 4 | 4 | 12 | 10 | 3 | 3 | 3 | 3 | 4 | 6 | 5 | 4 | 4 | 3 | 3 | 3 | 3 |
| M16B | mitochondrial processing peptidase beta-subunit | 5 | 5 | 9 | 10 | 8 | 9 | 6 | 4 | 6 | 13 | 6 | 5 | 5 | 5 | 5 | 9 | 5 | 5 | 5 | 3 | 5 | 5 | 5 |
| M16C | Eupitrilysin | 2 | 2 | 3 | 3 | 3 | 4 | 2 | 1 | 2 | 8 | 2 | 2 | 2 | 2 | 2 | 4 | 2 | 2 | 2 | 2 | 2 | 2 | 4 |
| M17 | leucine aminopeptidase 3 | 0 | 0 | 0 | 3 | 3 | 0 | 0 | 0 | 0 | 11 | 0 | 0 | 0 | 0 | 0 | 2 | 0 | 0 | 0 | 0 | 0 | 0 | 3 |
| M18 | aminopeptidase I | 2 | 2 | 3 | 3 | 2 | 4 | 2 | 2 | 3 | 5 | 2 | 2 | 3 | 2 | 2 | 1 | 2 | 2 | 2 | 2 | 3 | 2 | 2 |
| M19 | membrane dipeptidase | 2 | 2 | 2 | 1 | 1 | 0 | 1 | 5 | 3 | 4 | 1 | 2 | 2 | 2 | 4 | 1 | 3 | 2 | 2 | 3 | 1 | 2 | 1 |
| M20A | glutamate carboxypeptidase | 4 | 5 | 2 | 0 | 5 | 1 | 7 | 6 | 8 | 0 | 1 | 1 | 2 | 2 | 3 | 0 | 2 | 2 | 2 | 5 | 2 | 1 | 0 |
| M20B | peptidase T | 0 | 0 | 0 | 0 | 0 | 0 | 1 | 0 | 0 | 0 | 0 | 0 | 0 | 0 | 0 | 0 | 0 | 0 | 0 | 0 | 0 | 0 | 0 |
| M20D | carboxypeptidase Ss1 | 7 | 4 | 4 | 6 | 4 | 3 | 6 | 10 | 3 | 7 | 1 | 1 | 3 | 3 | 1 | 6 | 2 | 0 | 1 | 1 | 2 | 2 | 8 |
| M20F | carnosine dipeptidase II | 2 | 3 | 3 | 5 | 0 | 2 | 0 | 2 | 0 | 10 | 2 | 2 | 2 | 2 | 2 | 3 | 2 | 2 | 3 | 2 | 2 | 2 | 3 |
| M20 | not assigned to subfamily | 2 | 2 | 0 | 0 | 0 | 0 | 2 | 1 | 2 | 0 | 0 | 0 | 0 | 0 | 1 | 0 | 1 | 1 | 1 | 1 | 0 | 0 | 0 |
| M22 |  | 0 | 0 | 0 | 0 | 3 | 0 | 5 | 0 | 6 | 0 | 0 | 0 | 0 | 0 | 0 | 0 | 0 | 0 | 0 | 0 | 0 | 0 | 0 |
| M23 | lysostaphin | 0 | 0 | 0 | 0 | 0 | 0 | 28 | 16 | 38 | 0 | 0 | 0 | 0 | 0 | 0 | 0 | 0 | 0 | 0 | 0 | 0 | 0 | 0 |
| M24A | methionyl aminopeptidase 1 | 4 | 7 | 3 | 5 | 0 | 2 | 4 | 4 | 3 | 9 | 2 | 3 | 3 | 3 | 4 | 4 | 3 | 2 | 2 | 3 | 3 | 3 | 4 |
| M24B | aminopeptidase P | 6 | 4 | 5 | 9 | 6 | 9 | 6 | 8 | 6 | 17 | 5 | 4 | 5 | 5 | 5 | 7 | 5 | 5 | 5 | 7 | 5 | 5 | 4 |
| M24 | not assigned to subfamily | 3 | 3 | 5 | 4 | 10 | 6 | 3 | 3 | 3 | 6 | 2 | 2 | 2 | 1 | 3 | 4 | 3 | 3 | 3 | 2 | 3 | 3 | 3 |
| M28A | aminopeptidase S | 2 | 3 | 4 | 1 | 1 | 2 | 1 | 0 | 1 | 0 | 4 | 3 | 3 | 4 | 0 | 1 | 3 | 0 | 0 | 5 | 3 | 0 | 2 |
| M28B | glutamate carboxypeptidase II | 2 | 2 | 2 | 4 | 2 | 3 | 2 | 7 | 2 | 5 | 2 | 2 | 2 | 2 | 2 | 3 | 2 | 2 | 2 | 3 | 2 | 2 | 4 |
| M28E | aminopeptidase Ap1 | 2 | 1 | 3 | 2 | 0 | 2 | 1 | 1 | 0 | 2 | 2 | 2 | 2 | 2 | 1 | 1 | 1 | 1 | 1 | 1 | 1 | 2 | 1 |
| M28 | not assigned to subfamily | 1 | 0 | 6 | 3 | 3 | 5 | 2 | 3 | 1 | 9 | 4 | 5 | 4 | 2 | 2 | 4 | 1 | 2 | 2 | 1 | 3 | 3 | 4 |
| M35 | Deuterolysin | 0 | 3 | 0 | 0 | 1 | 0 | 1 | 0 | 0 | 0 | 0 | 0 | 0 | 0 | 0 | 0 | 1 | 0 | 0 | 0 | 1 | 0 | 0 |
| M36 | Fungalysin | 1 | 1 | 0 | 2 | 0 | 0 | 0 | 2 | 0 | 3 | 0 | 0 | 0 | 0 | 0 | 1 | 0 | 0 | 0 | 1 | 0 | 0 | 1 |
| M38 | isoaspartyl dipeptidase | 17 | 9 | 16 | 9 | 20 | 10 | 16 | 13 | 14 | 15 | 6 | 10 | 11 | 11 | 12 | 9 | 14 | 6 | 6 | 5 | 8 | 11 | 11 |
| M41 | FtsH peptidase | 2 | 2 | 2 | 4 | 16 | 3 | 2 | 4 | 2 | 6 | 2 | 2 | 2 | 2 | 2 | 5 | 2 | 2 | 2 | 2 | 2 | 2 | 5 |
| M42 | glutamyl aminopeptidase | 0 | 0 | 1 | 0 | 0 | 2 | 0 | 0 | 0 | 0 | 0 | 1 | 1 | 1 | 0 | 1 | 0 | 0 | 0 | 0 | 0 | 0 | 0 |
| M43B | Cytophagalysin | 1 | 1 | 1 | 0 | 1 | 1 | 1 | 0 | 1 | 0 | 1 | 1 | 1 | 1 | 1 | 0 | 1 | 1 | 1 | 2 | 0 | 0 | 0 |
| M48A | Ste24 peptidase | 1 | 1 | 1 | 2 | 1 | 2 | 1 | 1 | 1 | 1 | 1 | 1 | 1 | 1 | 1 | 1 | 1 | 1 | 1 | 1 | 1 | 1 | 1 |
| M48B | HtpX peptidase | 0 | 0 | 0 | 0 | 1 | 0 | 0 | 0 | 0 | 0 | 0 | 0 | 0 | 0 | 0 | 0 | 0 | 0 | 0 | 0 | 0 | 0 | 0 |
| M48C | Oma1 peptidase | 1 | 1 | 4 | 1 | 1 | 2 | 0 | 0 | 0 | 6 | 1 | 1 | 1 | 1 | 1 | 2 | 1 | 1 | 1 | 1 | 1 | 1 | 1 |
| M48 | not assigned to subfamily | 0 | 0 | 0 | 0 | 1 | 2 | 1 | 2 | 0 | 0 | 0 | 0 | 0 | 0 | 0 | 0 | 0 | 0 | 0 | 0 | 0 | 0 | 0 |
| M49 | dipeptidyl-peptidase III | 2 | 2 | 1 | 0 | 1 | 2 | 1 | 1 | 3 | 2 | 1 | 1 | 1 | 1 | 1 | 1 | 1 | 1 | 1 | 1 | 2 | 2 | 0 |
| M50 |  | 0 | 0 | 0 | 0 | 5 | 0 | 0 | 1 | 0 | 0 | 0 | 0 | 0 | 0 | 0 | 0 | 0 | 0 | 0 | 0 | 0 | 0 | 0 |
| M50B | sporulation factor SpoIVFB | 0 | 0 | 0 | 0 | 4 | 0 | 0 | 0 | 0 | 0 | 0 | 0 | 0 | 0 | 0 | 0 | 0 | 0 | 0 | 0 | 0 | 0 | 0 |
| M50A | site 2 peptidase | 0 | 0 | 0 | 1 | 0 | 0 | 0 | 2 | 0 | 2 | 0 | 0 | 0 | 0 | 0 | 1 | 0 | 0 | 0 | 0 | 0 | 0 | 1 |
| M54 | Archaelysin | 0 | 0 | 1 | 0 | 1 | 0 | 0 | 0 | 0 | 0 | 0 | 0 | 0 | 0 | 0 | 0 | 1 | 0 | 0 | 0 | 0 | 1 | 0 |
| M67A | RPN11 peptidase | 4 | 4 | 6 | 11 | 3 | 6 | 4 | 6 | 4 | 11 | 4 | 5 | 4 | 4 | 4 | 7 | 4 | 5 | 4 | 4 | 4 | 5 | 7 |
| M67C | STAMBP isopeptidase | 1 | 1 | 1 | 2 | 1 | 0 | 1 | 2 | 0 | 4 | 1 | 1 | 1 | 1 | 1 | 2 | 1 | 1 | 1 | 1 | 1 | 1 | 2 |
| M67 | not assigned to subfamily | 2 | 2 | 2 | 3 | 3 | 3 | 2 | 2 | 4 | 3 | 2 | 2 | 2 | 2 | 2 | 2 | 2 | 2 | 2 | 2 | 2 | 2 | 3 |
| M76 | Atp23 peptidase | 1 | 1 | 1 | 2 | 1 | 2 | 1 | 2 | 1 | 2 | 1 | 1 | 1 | 1 | 1 | 2 | 1 | 1 | 1 | 1 | 1 | 1 | 2 |
| M77 | tryptophanyl aminopeptidase 7-DMATS-type peptidase | 2 | 7 | 1 | 0 | 4 | 4 | 1 | 0 | 1 | 0 | 0 | 0 | 0 | 0 | 1 | 0 | 1 | 2 | 2 | 0 | 0 | 0 | 0 |
| M79 | RCE1 peptidase (Saccharomyces cerevisiae) | 1 | 1 | 1 | 0 | 5 | 2 | 0 | 0 | 0 | 2 | 1 | 1 | 1 | 1 | 1 | 1 | 1 | 1 | 1 | 1 | 1 | 1 | 1 |
| M80 | Wss1 peptidase | 3 | 2 | 3 | 1 | 2 | 2 | 0 | 0 | 0 | 4 | 2 | 3 | 3 | 3 | 2 | 2 | 2 | 2 | 2 | 3 | 3 | 3 | 2 |
| M81 | microcystinase MlrC | 1 | 0 | 0 | 0 | 1 | 0 | 0 | 0 | 0 | 0 | 0 | 0 | 0 | 0 | 0 | 0 | 0 | 1 | 1 | 0 | 0 | 0 | 0 |
| P2B | polycystin-1 | 0 | 0 | 0 | 0 | 0 | 0 | 0 | 0 | 0 | 0 | 0 | 0 | 0 | 0 | 0 | 0 | 0 | 0 | 0 | 0 | 1 | 0 | 0 |
| P1 | DmpA aminopeptidase | 0 | 1 | 1 | 0 | 0 | 0 | 0 | 0 | 0 | 0 | 0 | 0 | 0 | 0 | 0 | 0 | 0 | 0 | 0 | 0 | 0 | 0 | 0 |
| N9 | intein-containing V-type proton ATPase catalytic subunit A | 0 | 0 | 0 | 0 | 1 | 0 | 0 | 0 | 0 | 0 | 0 | 0 | 0 | 0 | 0 | 0 | 0 | 0 | 0 | 2 | 0 | 0 | 0 |
| N11 | intein-containing chloroplast ATP-dependent peptide lyase | 0 | 0 | 0 | 0 | 3 | 0 | 0 | 0 | 00 | 0 | 0 | 0 | 0 | 0 | 0 | 0 | 0 | 0 | 0 | 0 | 0 | 0 | 0 |
| S1A | chymotrypsin A | 0 | 0 | 0 | 1 | 14 | 1 | 0 | 2 | 1 | 2 | 0 | 0 | 0 | 0 | 0 | 1 | 0 | 0 | 0 | 0 | 0 | 0 | 0 |
| S1B | glutamyl endopeptidase I | 0 | 0 | 0 | 0 | 0 | 0 | 1 | 3 | 5 | 0 | 0 | 0 | 0 | 0 | 0 | 0 | 0 | 0 | 0 | 0 | 0 | 0 | 0 |
| S1C | DegP peptidase | 0 | 0 | 0 | 0 | 1 | 0 | 0 | 0 | 0 | 0 | 1 | 0 | 0 | 0 | 0 | 0 | 0 | 0 | 0 | 0 | 0 | 0 | 0 |
| S1D | lysyl endopeptidase | 1 | 1 | 1 | 4 | 0 | 3 | 0 | 1 | 0 | 6 | 1 | 1 | 1 | 1 | 2 | 3 | 2 | 1 | 1 | 1 | 1 | 1 | 3 |
| S1E | streptogrisin A | 0 | 0 | 0 | 0 | 0 | 0 | 0 | 0 | 0 | 0 | 0 | 0 | 0 | 0 | 0 | 0 | 1 | 0 | 0 | 0 | 0 | 0 | 0 |
| S3 | Togavirin | 0 | 0 | 0 | 0 | 0 | 2 | 0 | 0 | 0 | 0 | 0 | 0 | 0 | 0 | 0 | 0 | 0 | 1 | 0 | 0 | 0 | 0 | 0 |
| S8A | subtilisin Carlsberg | 5 | 4 | 20 | 31 | 12 | 20 | 2 | 15 | 9 | 36 | 7 | 8 | 9 | 9 | 2 | 17 | 2 | 1 | 1 | 9 | 8 | 8 | 18 |
| S8B | Kexin | 1 | 1 | 2 | 3 | 0 | 2 | 2 | 2 | 1 | 3 | 1 | 1 | 1 | 1 | 2 | 2 | 1 | 1 | 1 | 1 | 1 | 1 | 2 |
| S9A | prolyl oligopeptidase | 0 | 0 | 0 | 2 | 4 | 0 | 0 | 1 | 0 | 2 | 0 | 0 | 0 | 0 | 0 | 1 | 0 | 0 | 0 | 0 | 0 | 0 | 1 |
| S9B | dipeptidyl-peptidase IV | 1 | 4 | 5 | 4 | 4 | 6 | 1 | 4 | 5 | 7 | 4 | 4 | 4 | 3 | 0 | 2 | 1 | 2 | 1 | 3 | 2 | 1 | 2 |
| S9C | acylaminoacyl-peptidase | 22 | 11 | 15 | 10 | 4 | 12 | 1 | 2 | 3 | 10 | 6 | 8 | 7 | 8 | 7 | 12 | 8 | 7 | 7 | 8 | 11 | 9 | 8 |
| S9 |  | 69 | 39 | 52 | 21 | 175 | 45 | 54 | 29 | 52 | 33 | 21 | 20 | 24 | 27 | 30 | 23 | 37 | 17 | 16 | 36 | 19 | 29 | 10 |
| S10 | carboxypeptidase Y | 14 | 12 | 7 | 8 | 12 | 7 | 11 | 5 | 8 | 11 | 3 | 4 | 6 | 6 | 5 | 4 | 9 | 4 | 4 | 3 | 3 | 5 | 4 |
| S12 | D-Ala-D-Ala carboxypeptidase B | 10 | 6 | 11 | 0 | 18 | 10 | 11 | 1 | 17 | 2 | 1 | 1 | 5 | 5 | 6 | 1 | 7 | 1 | 1 | 5 | 2 | 7 | 2 |
| S13 | D-Ala-D-Ala peptidase C | 0 | 0 | 0 | 0 | 0 | 0 | 0 | 0 | 0 | 0 | 0 | 0 | 0 | 0 | 0 | 0 | 0 | 0 | 0 | 1 | 0 | 0 | 0 |
| S15 | Xaa-Pro dipeptidyl-peptidase | 0 | 0 | 0 | 0 | 2 | 0 | 3 | 0 | 3 | 0 | 0 | 0 | 0 | 0 | 0 | 0 | 0 | 0 | 0 | 0 | 0 | 0 | 0 |
| S14 | peptidase Clp | 1 | 1 | 2 | 1 | 1 | 1 | 1 | 1 | 1 | 2 | 1 | 1 | 1 | 1 | 1 | 1 | 1 | 1 | 1 | 1 | 1 | 1 | 1 |
| S16 | Lon-A peptidase | 2 | 2 | 4 | 6 | 7 | 1 | 3 | 4 | 2 | 6 | 2 | 2 | 2 | 2 | 1 | 6 | 2 | 2 | 2 | 2 | 2 | 2 | 5 |
| S26A | signal peptidase I | 2 | 2 | 3 | 0 | 3 | 2 | 2 | 2 | 2 | 3 | 1 | 2 | 2 | 1 | 2 | 2 | 2 | 2 | 2 | 2 | 1 | 2 | 2 |
| S26B | signalase 21 kDa componente | 1 | 1 | 2 | 1 | 0 | 1 | 1 | 1 | 1 | 0 | 1 | 1 | 1 | 1 | 2 | 1 | 2 | 1 | 1 | 2 | 1 | 1 | 1 |
| S28 | lysosomal Pro-Xaa carboxypeptidase | 3 | 3 | 5 | 1 | 1 | 9 | 1 | 1 | 5 | 4 | 2 | 2 | 3 | 3 | 3 | 2 | 0 | 2 | 2 | 0 | 4 | 2 | 0 |
| S29 | Hepacivirin | 0 | 0 | 0 | 0 | 7 | 0 | 0 | 0 | 0 | 0 | 0 | 0 | 0 | 0 | 0 | 0 | 0 | 0 | 0 | 0 | 0 | 0 | 0 |
| S33 | prolyl aminopeptidase | 60 | 41 | 54 | 22 | 64 | 39 | 37 | 21 | 49 | 43 | 23 | 25 | 32 | 31 | 38 | 22 | 29 | 17 | 17 | 21 | 27 | 30 | 13 |
| S41A | C-terminal processing peptidase-1 | 0 | 0 | 0 | 0 | 1 | 0 | 0 | 1 | 0 | 1 | 0 | 0 | 0 | 0 | 0 | 0 | 0 | 0 | 0 | 0 | 0 | 0 | 0 |
| S49B | protein C | 0 | 0 | 0 | 0 | 0 | 2 | 0 | 0 | 0 | 0 | 0 | 0 | 0 | 0 | 0 | 0 | 0 | 0 | 0 | 0 | 0 | 0 | 0 |
| S49C | archaean signal peptide peptidase 1 | 0 | 0 | 0 | 0 | 14 | 0 | 0 | 0 | 0 | 0 | 0 | 0 | 0 | 0 | 0 | 0 | 0 | 0 | 0 | 0 | 0 | 0 | 0 |
| S51 | dipeptidase E | 0 | 0 | 0 | 0 | 1 | 0 | 0 | 0 | 0 | 0 | 0 | 0 | 0 | 0 | 0 | 0 | 0 | 0 | 0 | 0 | 0 | 0 | 0 |
| S53 | Sedolisin | 4 | 5 | 7 | 0 | 5 | 6 | 4 | 1 | 12 | 0 | 5 | 2 | 6 | 3 | 8 | 0 | 3 | 2 | 0 | 5 | 2 | 7 | 0 |
| S54 | rhomboid-1 | 3 | 3 | 3 | 6 | 21 | 6 | 2 | 8 | 2 | 8 | 3 | 2 | 3 | 3 | 4 | 5 | 3 | 3 | 3 | 3 | 3 | 3 | 5 |
| S58 |  | 0 | 0 | 0 | 0 | 0 | 0 | 0 | 0 | 1 | 0 | 0 | 0 | 0 | 0 | 0 | 0 | 0 | 0 | 0 | 0 | 0 | 0 | 0 |
| S59 | nucleoporin 145 | 1 | 1 | 1 | 1 | 1 | 1 | 1 | 1 | 1 | 2 | 1 | 1 | 1 | 1 | 1 | 1 | 1 | 1 | 1 | 1 | 1 | 1 | 1 |
| S64 | Ssy5 peptidase | 0 | 0 | 0 | 0 | 5 | 0 | 0 | 0 | 0 | 0 | 0 | 0 | 0 | 0 | 0 | 0 | 0 | 0 | 0 | 0 | 0 | 0 | 0 |
| S66 | murein tetrapeptidase LD-carboxypeptidase | 1 | 0 | 0 | 0 | 0 | 0 | 0 | 0 | 0 | 0 | 0 | 0 | 0 | 0 | 0 | 0 | 0 | 0 | 0 | 0 | 0 | 0 | 0 |
| S79 | CARD8 self-cleaving protein | 0 | 0 | 0 | 0 | 1 | 0 | 0 | 0 | 0 | 0 | 0 | 0 | 0 | 0 | 0 | 0 | 0 | 0 | 0 | 0 | 0 | 0 | 0 |
| T1A | archaean proteasome, beta componente | 11 | 12 | 13 | 23 | 3 | 15 | 15 | 14 | 16 | 20 | 11 | 11 | 11 | 12 | 12 | 11 | 12 | 11 | 11 | 11 | 11 | 11 | 14 |
| T1 | not assigned to subfamily | 3 | 3 | 6 | 5 | 2 | 5 | 1 | 0 | 1 | 4 | 5 | 6 | 4 | 6 | 3 | 3 | 4 | 4 | 4 | 5 | 5 | 5 | 3 |
| T2 | glycosylasparaginase precursor | 2 | 3 | 8 | 1 | 2 | 2 | 1 | 2 | 6 | 3 | 6 | 3 | 7 | 6 | 3 | 1 | 3 | 3 | 3 | 6 | 6 | 6 | 1 |
| T3 | gamma-glutamyltransferase 1 | 5 | 3 | 3 | 4 | 11 | 1 | 3 | 4 | 81 | 10 | 3 | 2 | 3 | 3 | 5 | 2 | 3 | 3 | 3 | 5 | 3 | 3 | 3 |
| T5 | ornithine acetyltransferase precursor | 1 | 1 | 1 | 1 | 1 | 2 | 1 | 1 | 1 | 2 | 1 | 1 | 1 | 1 | 1 | 1 | 1 | 1 | 1 | 1 | 1 | 1 | 1 |
| T7 | CwpV self-cleaving threonine peptidase | 0 | 0 | 0 | 0 | 8 | 0 | 0 | 0 | 0 | 0 | 0 | 0 | 0 | 0 | 0 | 0 | 0 | 0 | 0 | 0 | 0 | 0 | 0 |
| U32 | collagenase | 0 | 0 | 0 | 0 | 5 | 0 | 0 | 0 | 0 | 0 | 0 | 0 | 0 | 0 | 0 | 0 | 0 | 0 | 0 | 0 | 0 | 0 | 0 |
| U48 |  | 0 | 0 | 0 | 0 | 0 | 1 | 1 | 0 | 1 | 0 | 0 | 0 | 0 | 0 | 0 | 0 | 0 | 0 | 0 | 0 | 0 | 0 | 0 |
| U69 | AIDA-I self-cleaving autotransporter protein | 0 | 0 | 0 | 0 | 5 | 0 | 0 | 0 | 0 | 0 | 0 | 0 | 0 | 0 | 0 | 0 | 0 | 0 | 0 | 0 | 0 | 0 | 0 |

**Additional file 3.** Peptidase families that most contribute (Cumulative >50% of contribution) to the differences between thermophilic and mesophilic fungi as shown by Percentage of similarity analysis (SIMPER).

| **Family** | **Enzyme type** | **Contrib. %** | **Cumulative %** |
| --- | --- | --- | --- |
| S9 | prolyl oligopeptidase | 12.58 | 12.58 |
| S33 | prolyl aminopeptidase | 6.87 | 19.45 |
| A11A | Copia transposon peptidase | 6.34 | 25.79 |
| A1A | pepsin A | 3.89 | 29.67 |
| M23 | beta-lytic metallopeptidase | 3.40 | 33.07 |
| S8A | Subtilisin | 3.37 | 36.44 |
| T3 | gamma-glutamyltransferase 1 | 3.30 | 39.74 |
| C19 | ubiquitin-specific peptidase | 2.92 | 42.66 |
| S12 | D-Ala-D-Ala carboxypeptidase B | 2.54 | 45.21 |
| M13 | neprilysin | 2.39 | 47.60 |
| S9C | acylaminoacyl-peptidase | 2.16 | 49.76 |

**Additional file 4.** Differences between the number of cavities in peptidases from mesophilic and thermophilic fungi. The differences were tested using the T-test, with *n*-1 degrees of freedom, for a total of 102,521 and 114,946 proteins and 3,590 and 3,340 peptidases from mesophilic and thermophilic fungi, respectively.

| **Amino Acid** | **Proteins** | |  |  | **Peptidases** | |  |  |
| --- | --- | --- | --- | --- | --- | --- | --- | --- |
|  | **Mesophilic (%)** | **Thermophilic (%)** | ***P*-value** | **T-value** | **Mesophilic (%)** | **Thermophilic (%)** | ***P*-value** | **T-value** |
| Alanine (Ala) | 8.11±2.75 | 9.06±2.80 | 0 | -79.534 | 8.34±2.05 | 8.99±2.12 | 3.8375E-34 | -12.9910 |
| Cysteine (Cys) | 1.45±1.30 | 1.38±1.28 | 5.5243E-36 | 12.526 | 1.27±0.84 | 1.22±0.82 | 0.030096 | 2.1693 |
| Aspartic acid (Asp) | 5.49±1.98 | 5.44±2.01 | 3.1899E-07 | 5.112 | 5.85±1.39 | 5.84±1.30 | 0.81496 | 0.23404 |
| Glutamine (Glu) | 6.06±2.57 | 6.21±2.63 | 1.7037E-36 | -12.619 | 6.04±1.97 | 6.14±1.84 | 0.023085 | -2.2725 |
| Phenylalanine (Phe) | 3.79±1.68 | 3.54±1.66 | 1.4974E-252 | 33.989 | 3.85±1.24 | 3.82±1.15 | 0.38783 | 0.8636 |
| Glycine (Gly) | 6.50±2.68 | 7.10±2.60 | 0 | -52.725 | 7.35±2.03 | 7.88±2.00 | 1.4010E-27 | -10.9290 |
| Histidine (His) | 2.50±1.33 | 2.39±1.27 | 1.3166E-89 | 20.081 | 2.55±0.99 | 2.50±0.89 | 0.028816 | 2.1864 |
| Isoleucine (Ile) | 5.21±2.01 | 4.51±1.90 | 0 | 83.41 | 5.28±1.50 | 4.76±1.36 | 2.4014E-51 | 15.1990 |
| Lysine (Lys) | 5.27±2.70 | 4.79±2.56 | 0 | 42.824 | 4.95±1.93 | 4.61±1.74 | 8.8003E-15 | 7.7726 |
| Leucine (Leu) | 8.91±2.60 | 8.73±2.61 | 2.6382E-57 | 15.959 | 8.90±1.87 | 8.90±1.81 | 0.98962 | -0.013016 |
| Methionine (Met) | 2.35±1..12 | 1.27±1.16 | 1.3336E-303 | 37.303 | 2.11±0.88 | 2.02±0.82 | 2.2406E-05 | 4.2424 |
| Asparagine (Asn) | 3.99±1.73 | 3.42±1.52 | 0 | 81.95 | 4.06±1.37 | 3.57±1.19 | 5.3008E-55 | 15.7590 |
| Proline (Pro) | 5.69±2.50 | 6.35±2.69 | 0 | -58.794 | 5.51±1.78 | 5.93±1.81 | 3.6919E-22 | -9.7125 |
| Glutamine (Gln) | 4.11±1.91 | 3.96±1.94 | 2.2636E-67 | 17.348 | 3.83±1.35 | 3.57±1.27 | 1.1243E-16 | 8.3118 |
| Arginine (Arg) | 5.95±2.44 | 6.88±2.64 | 0 | -85.223 | 5.22±1.78 | 5.73±1.74 | 2.0930E-33 | -12.1070 |
| Serine (Ser) | 8.02±2.83 | 7.94±2.91 | 7.8103E-22 | 6.5045 | 7.64±1.97 | 7.30±1.88 | 1.4915E-13 | 7.4028 |
| Threonine (Thr) | 6.02±2.07 | 5.76±1.99 | 1.7707E-186 | 29.153 | 6.07±1.62 | 5.82±1.49 | 8.2074E-11 | 6.5069 |
| Valine (Val) | 6.14±2.10 | 6.20±2.02 | 3.7441E-10 | -6.2646 | 6.45±1.57 | 6.75±1.50 | 2.0993E-11 | -7.9537 |
| Tryptophan (Trp) | 1.44±1.01 | 1.43±1.27 | 0.0033329 | 2.9353 | 1.48±0.84 | 1.43±0.79 | 0.030897 | 2.1588 |
| Tyrosine (Tyr) | 2.99±1.47 | 2.73±1.48 | 0 | 40.976 | 3.28±1.21 | 3.23±1.13 | 0.070056 | 1.8118 |
| Charged | 25.28±5.86 | 25.72±5.87 | 5.2468E-67 | -17.3 | 24.60±4.55 | 24.82±4.18 | 0.039755 | -2.0567 |
| Polar | 23.58±4.78 | 22.46±4.73 | 0 | 54.739 | 22.86±3.68 | 21.48±3.52 | 3.2677E-56 | 15.9410 |
| Hydrophobic | 44.64±5.61 | 44.73±5.67 | 0.0004129 | -3.5337 | 45.19±3.90 | 45.83±3.60 | 2.4498E-12 | -7.0188 |

**Additional file 5.** Validation parameters computed for built 3D protein of the Aspartic peptidase sequence.

| **Fungi** | **QMEAN** | **Z-score** | **Lgscore** | **Ramachandran (%)*** | | | |
| --- | --- | --- | --- | --- | --- | --- | --- |
|  |  |  |  | **MFR** | **AAR** | **GAR** | **DR** |
| *Aspergillus fumigatus* | 0.54 | -9.09 | 5.179 | 86.4 | 10.2 | 2.7 | 0.7 |
| *Aspergillus niger* | 0.6 | -7.73 | 4.454 | 84.7 | 12.3 | 2.7 | 0.3 |
| *Chaetomium globosum* | 0.32 | -7.16 | 5.878 | 86.6 | 10.7 | 2.4 | 0.3 |
| *Chaetomium thermophilum* | 0.53 | -7.92 | 5.539 | 84.6 | 11.9 | 3.1 | 0.3 |
| *Myceliophthora fergusii* | 0.5 | -7.18 | 5.853 | 87.5 | 9.5 | 2.7 | 0.3 |
| *Myceliophthora sepedonium* | 0.5 | -6.87 | 5.887 | 85.6 | 11.3 | 2.7 | 0.3 |
| *Myceliophthora thermophila* | 0.5 | -7.18 | 5.853 | 87.5 | 9.5 | 2.7 | 0.3 |
| *Myriococcum thermophilum* | 0.49 | -7.66 | 5.791 | 85.5 | 11.4 | 2.4 | 0.7 |
| *Rasamsonia byssochlamydoides* | 0.59 | -8.47 | 5.154 | 85.4 | 11.8 | 1.7 | 1.0 |
| *Thermoascus crustaceus* | 0.58 | -8.1 | 5.052 | 88.4 | 9.9 | 1.4 | 0.3 |
| *Thielavia australiensis* | 0.55 | -6.8 | 5.893 | 87.8 | 8.7 | 2.8 | 0.7 |
| *Thielavia terrestris* | 0.34 | -7.5 | 5.566 | 85.5 | 11.8 | 2.8 | 0 |

* MFR: Most Favored Region; AAR: Additionally Allowed Region; GAR: Generally Allowed Region; DR: Disallowed Region.

**Additional file 6.** Predicted three-dimensional structures of selected aspartic acid peptidases of fungi. (A) *Aspergillus fumigatus*; (B) *A. niger*; (C) *Chaetomim globosum*; (D) *C. thermophilum*; (E) *Myceliophthora fergusii*; (F) *M. sepedonium*; (G) *M. thermophila*; (H) *Myriococcum thermophilum*; (I) *Rasamsonia bycochlamydoides*; (J) *Thermoascus crustaceus*; (K) *Thielavia australiensis*; and (L) *T. terrestris*.


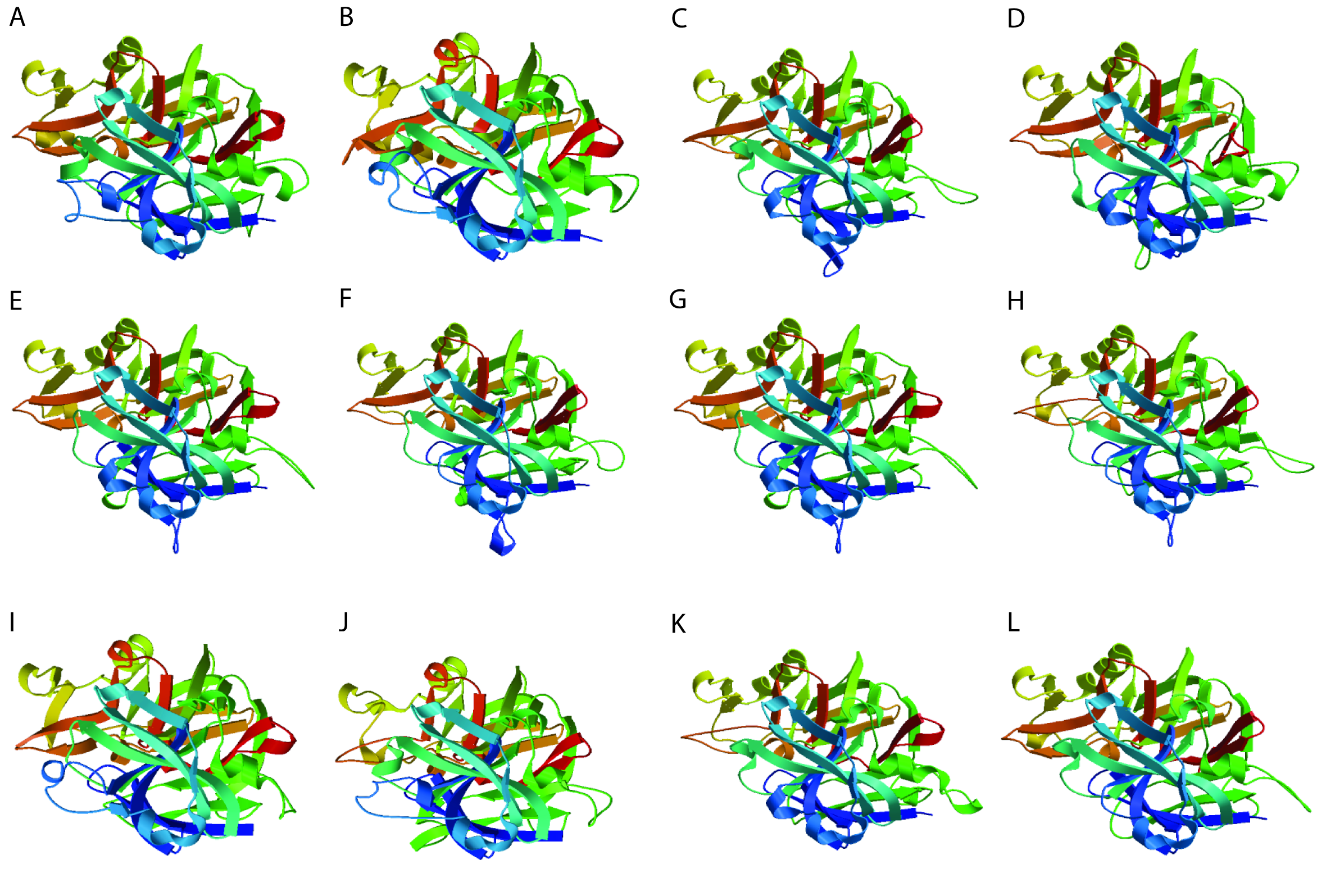

Supplement: Supplementary file 1 — Table S1 Contribution of the seven catalytic types for the differences between peptidases of thermophilic and mesophilic species as shown by Percentage of similarity analysis (SIMPER). Table S2 Catalogue of peptidases in thermophilic, thermotolerant and mesophilic fungal genomes. Table S3 Peptidase families that most contribute (Cumulative > 50% of contribution) to the differences between thermophilic and mesophilic fungi as shown by analysis of percentage of similarity (SIMPER). Table S4 Differences between the number of cavities in peptidases from mesophilic and thermophilic fungi. The differences were tested using the T-test, with n-1 degrees of freedom, for a total of 102,521 and 114,946 proteins and 3590 and 3340 peptidases from mesophilic and thermophilic fungi, respectively. Table S5 Validation parameters computed for built 3D protein of the Aspartic peptidase sequence. Figure S1 Predicted three-dimmensional structures of selected aspartic acid peptidases of fungi. (A) Aspergillus fumigatus; (B) A. niger; (C) Chaetomim globosum; (D) C. thermophilum; (E) Myceliophthora fergusii; (F) M. sepedonium; (G) M. thermophila; (H) Myriococcum thermophilum; (I) Rasamsonia bycochlamydoides; (J) Thermoascus crustaceus; (K) Thielavia australiensis; and (L) T. terrestris. (DOCX 3863 kb) [file 12864_2018_4549_MOESM1_ESM.docx]
